# Motor function in type 2 and 3 SMA patients treated with Nusinersen: a critical review and meta-analysis

**DOI:** 10.1186/s13023-021-02065-z

**Published:** 2021-10-13

**Authors:** Giorgia Coratti, Costanza Cutrona, Maria Carmela Pera, Francesca Bovis, Marta Ponzano, Fabrizia Chieppa, Laura Antonaci, Valeria Sansone, Richard Finkel, Marika Pane, Eugenio Mercuri

**Affiliations:** 1 Pediatric Neurology, Catholic University of Sacred Heart, Largo Gemelli 8, 00168 Rome, Italy; 2 Centro Clinico Nemo, Fondazione Policlinico Universitario Agostino Gemelli IRCCS, Rome, Italy; 3 Biostatistics Unit, Department of Health Sciences, University of Genoa, Genoa, Italy; 4 Neurorehabilitation Unit, Neuromuscular Omnicentre Clinical Center, Niguarda Hospital, University of Milan, Milan, Italy; 5 St. Jude Children’s Research Hospital, Memphis, USA

**Keywords:** Spinal muscular atrophy, Critical review, Nusinersen

## Abstract

**Background:**

There is an increasing number of papers reporting the real world use of Nusinersen in different cohorts of SMA patients.

**Main body:**

The aim of this paper was to critically review the literature reporting real world data on motor function in type 2 and 3 patients treated with Nusinersen, subdividing the results according to SMA type, age and type of assessment and performing a meta-analysis of the available results. We also report the available data collected in untreated patients using the same measures. Of the 400 papers identified searching for Nusinersen and spinal muscular atrophy, 19 reported motor function in types 2 and 3: 13 in adults, 4 in children and 2 included both. Twelve papers reported untreated patients’ data. All studies reported positive changes on at least one of the functional measures and at every time point while all-untreated cohorts showed negative changes.

**Conclusion:**

Our review suggests that Nusinersen provides a favorable benefit in motor function across a wide range of SMA type 2 and 3 patients over a 10–14 month observation period. Although a direct comparison with studies reporting data from untreated patients cannot be made, the longitudinal changes in the treated cohorts (consistently positive) are divergent from those observed in the untreated cohorts (consistently negative). The difference could be observed both in the global cohorts and in smaller groups subdivided according to age, type or functional status.

**Supplementary Information:**

The online version contains supplementary material available at 10.1186/s13023-021-02065-z.

## Background

Spinal muscular atrophy (SMA) is an autosomal recessive genetic disease characterized by degeneration of the α-motor neurons of the anterior horn cells of the brain stem and spinal cord, leading to progressive muscle weakness [1]. SMA is caused by mutations in the survival motor neuron 1 (*SMN1*) gene, encoding the SMN protein, which is essential for motor neuron survival. A limited amount of functional SMN protein is produced by another gene, *SMN2*, also located on chromosome 5q. *SMN2* differs from *SMN1* by few nucleotides, one of which creates an alternative splicing motif in exon 7 that largely exclude it from the mature *SMN2* mRNA.

In the last few years a number of therapeutic approaches have targeted a possible increase of the production of SMN protein in target motor neurons by genetic replacement of the defective *SMN1* gene [2, 3] or by modifying pre-mRNA splicing in *SMN2* to promote exon 7 inclusion by using an antisense oligonucleotide or small molecule drugs [4–7].

Nusinersen, an antisense oligonucleotide that targets pre-mRNA splicing of the SMN2 gene, is the first medical treatment approved for SMA. Following two successful pivotal trials in early-infantile and later-infantile onset SMA [6, 7], the drug was first approved by Food and Drug Administration (FDA) in 2016 and over the following years by the and by European Medicines Agency (EMA) and several other countries worldwide.

Most of the early real-world data have focused on type 1 infants enrolled in early access programs, [8–10] but in the last few years several studies have reported additional data in older children [11–14] and adults [15–29], covering the whole spectrum of SMA, from young infants with the most severe forms [30] to adults with a milder phenotype. The real-world data have expanded our knowledge on safety and efficacy of the drug in a much larger population of SMA patients than those reported in the pivotal studies. As of mid-2021 over 11,000 patients with SMA have been treated with Nusinersen [31].

The papers reporting the use of Nusinersen in infants with early onset SMA consistently showed an increase in survival and function and achievement of motor milestones [8–10]. These findings are different from the known natural history of untreated type 1 infants who had reduced survival and never showed any functional improvement [32–36]. Functional improvement has also been reported in children and adult classically labeled as type 2 and 3 but the interpretation of the data and the comparison between different datasets is complicated by the differences in the cohorts studied and by the tools used to establish efficacy. The interpretation of the results is further complicated by the relative paucity of age specific reference data in untreated patients, especially in adult cohorts [26, 37–46]. The aim of this paper was to critically review the existing literature on motor function in type 2 and 3 patients treated with Nusinersen, trying to establish possible patterns of efficacy by subdividing the results according to SMA type, age (pediatric vs adults) and type of assessment. When available, for each measure, we also reported data collected in untreated patients using the same measures.

## Main text

### Search methodology

PRISMA guidelines were applied in order to conduct the critical review, including research on online-databases for peer-reviewed journal (PUBMED, MEDILINE, Web of Science, CINAHL, PsycINFO, and EMBASE), and manual research on reference lists of included articles. To identify articles on SMA motor trajectories in the field of nusinersen treatment, the following key terms were used: “Spinal Muscular Atrophy”, “SMA”, combined with keywords “Nusinersen”, “Spinraza”. In parallel, we also performed a search combing “SMA” or “Spinal Muscular Atrophy” with “Motor function”, “Outcome measures”, “natural history” to identify changes in untreated patients. All electronic searches were limited to the English language and to publication until Jan 2021. As the studies on nusinersen were all relatively recent, with the first studies performed approximately 10 years ago, we decided to include natural history data from the same decade.

The screening and data collection procedure was given by five authors (CC, FC, GC, LA, MCP). Full-text review was applied by the same group and by the senior author (EM) to determine the full eligibility of articles. Studies reporting data on type 1 only were excluded. Eligible articles reporting treated patients were grouped into categories based on SMA type (SMA 2, SMA 3, combination) or age (pediatric vs adult). A similar approach was used to classify data from untreated patients. When needed, re-calculation on mean and standard deviation of age or motor outcome was performed from papers reporting full data access.

### Statistical analysis

Data were analyzed looking at the reported changes and standard deviation (SD) in the individual motor outcome measures in the different treated groups, subdivided by age category (adults, pediatric), motor function (ambulant, non-ambulant) and SMA type [2, 3].

Data extracted included the name of the first author, outcome measure, target population (n, type, age category, age range at treatment, mean age at treatment), magnitude of changes at 10, 12 or 14 months from baseline (mean, standard deviation/95% confidence interval).

The pooled analysis was conducted at different levels: first a rough evaluation on the overall benefit of treatment vs no-treatment was run including the largest available evidence, even if heterogeneous. The effect size was estimated using random-effect models and heterogeneity among studies was quantified by the I^2^ coefficient [47].

Subgroup analyses were further conducted to verify and estimate the influence of different categories (age, SMA type and motor function) on the pooled results of the treated population.

Meta-regression analysis was undertaken in order to identify possible sources of heterogeneity among studies. Meta-regression analyses were employed with random-effects model using aggregate-level data. Only studies with complete data available (sample N, mean, standard deviation/95% confidence interval) were included in the meta-analysis.

The statistical software Stata (v.16; IBM Corp.) was to run the meta-analyses and draw the forest plots.

### Search results

Applying the search terminology, a total of 14,627 articles were preliminarily selected based on their title. Of this, 788 were related to nusinersen treatment. After reviewing the full abstracts, 9221 were excluded from the review of the full paper.

**Fig. 1:**
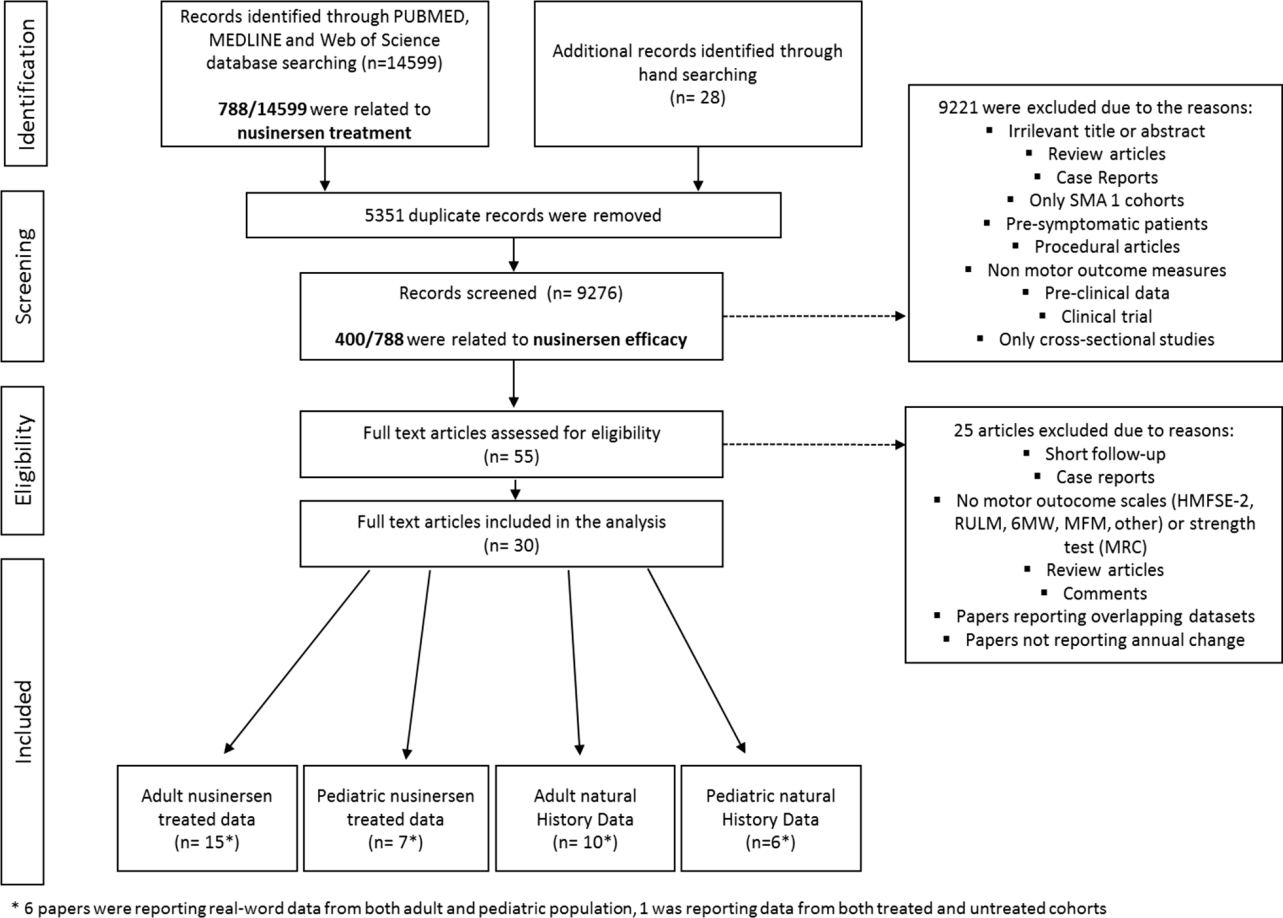
Search and selection process (PRISMA framework)

The review of the literature showed over 400 papers on Nusinersen. Approximately the 15% of these studies focused on modalities of administration or safety, other studies reported socioeconomic issues (7%), electrophysiology or biomarkers (6%). Others were review articles or general papers not reporting details of efficacy using functional scales or motor outcome measures. After reviewing the full papers, 30 papers were selected and analyzed. Figure 1 describes the search and selection process using the PRISMA framework.

After excluding reviews, commentaries or individual case reports (see Fig. 1 for details), we selected 19 papers reporting data on efficacy using structured assessments in type 2, 3. In 4 of the 19 papers type 1 patients were also included, with 1 of the 4 papers describing type 1 data separately. None of the HFMSE, RULM and 6MWT data included type 1 patients. Type 1 patients were only part of CHOP-INTEND, MFM and HINE 2 datasets. Data from clinical trials were not included in the review. The 19 papers were reviewed using a risk of bias assessment tool for non randomized studies (RoBANS) [48] (see Additional file 3: Fig. S1).

Twelve papers reported data in untreated patients.

#### Results on motor function scales

##### Hammersmith Functional Motor Scale Expanded (HFMSE)

**Fig. 2:**
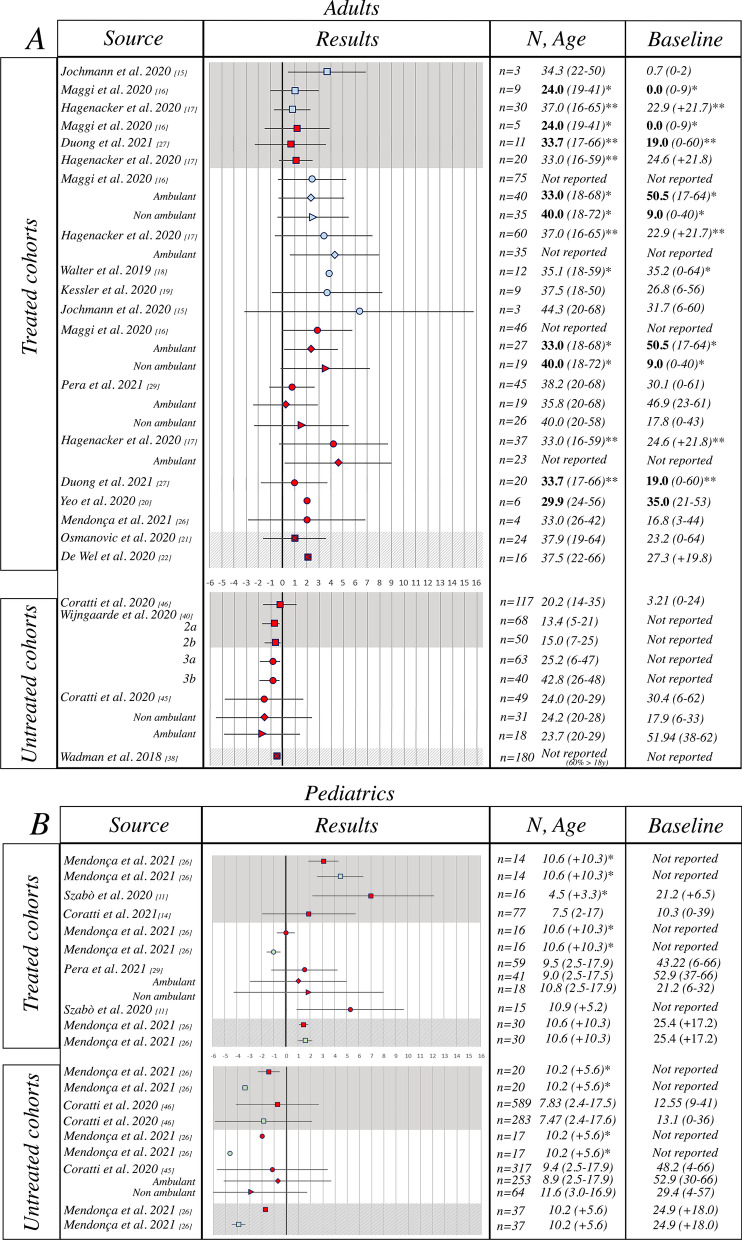
Hammersmith Functional Motor Scale Expanded results reporting author, HFMSE results (0–66 scale, mean ± SD), sample size, mean age (years, range/SD), mean baseline HFMSE (range/SD). **a** Adult population, **b** pediatric population. Key to figure: dashed line = 95% Confidence interval, plain line = Standard Deviation, square = SMA 2, circle = SMA 3, diamond = ambulant SMA 3, triangle = non ambulant SMA 3, square + circle + triangle = mix phenotypes. Bold font = Median value, Italic = Mean value. Color coding: light blue =  ~10 months from initiation of drug, Red =  ~12 months from initiation of drug, Green =  ~24 months from infusion. Grey shade = SMA 2, White shade = SMA 3, Striped shade = mixed phenotypes. *Mean/median values of the baseline population non excluding drop-outs at T10, 14 or 24 months of follow-up, **mean/median values of the baseline population of both SMA II and III combined

A total of 13 papers reporting nusinersen treated patients and 5 papers reporting studies from untreated cohorts were identified. Two of the 13 studies reporting nusinersen treated patients included both pediatric and adult patients, 9 only adults and 2 only pediatric patients (Fig. 2). Individual data on HFMSE were available in three papers (Mendonca 2020, Jochmann 2020 e Kessler 2019), therefore, in order to subgroup the populations according to type, age group or avoid missing data, we have re-calculated mean change overtime.

All the manuscripts describing nusinersen treated patients reported an increase on the HFMSE score and overall, the benefit of the treatment resulted to be statistically significant (pooled mean change = 2.27 (95% CI 1.41–3.13)). For the untreated patients the pooled mean change was − 1.00 (95% CI − 1.33; − 0.67) indicating a significant reduction of the HFMSE score from baseline. Pooled mean change across treated and untreated patients’ datasets was statistically significant (p < 0.0001).

Since heterogeneity was considerable (I^2^ = 90%) and highly significant we performed a multivariate meta-regression analysis. When adjusting by age group, SMA type and treatment, we found that the difference in the HFMSE score was associated with the nusinersen treatment (coefficient (standard error (SE)): 3.30 (0.51), p < 0.0001), while the HFMSE score change was not significantly associated with SMA type (2/3) (p = 0.437) and age group (adult/pediatric) (p = 0.981). Results remained consistent when we analyzed studies with 10, 12, 14 or 24 months of follow-up, separately (data not shown).

##### Subgroup analysis in treated patients

###### Adult and pediatric population

Both adult and pediatric populations reported an increase on the HFMSE score (pooled mean change = 1.87 (95% CI 1.05–2.68) and 2.98 (95% CI 0.97–4.99), respectively, with no difference in pooled mean change between the two populations (p = 0.320) (Additional file 4: Fig. S2).

###### SMA type

Both SMA type 2 and SMA type 3 populations reported a significant increase on the HFMSE score (pooled mean change = 2.54 (95% CI 1.00–4.09) and 2.26 (95% CI 1.06–3.47), respectively), with no difference in pooled mean change between the two populations (p = 0.780).

###### Ambulatory status

Both ambulant and non-ambulant populations reported a significant increase on the HFMSE score (pooled mean change = 1.99 (95% CI 0.24–3.74 and = 2.39 (95% CI 0.99–3.79), respectively), with no difference in pooled mean change between the two populations (p = 0.730).

#### Revised Upper Limb Module (RULM)

**Fig. 3:**
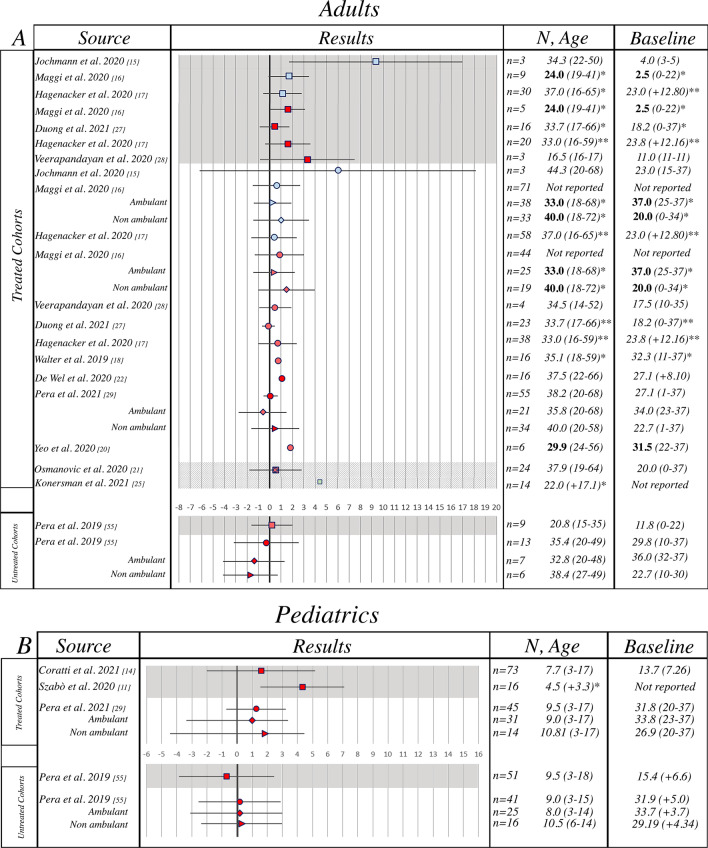
Revised Upper Limb Module results reporting author, results (mean ± SD), sample size, mean age (years, range/SD), mean baseline RULM (range/SD). **a** Adult population, **b** pediatric population. Key to figure: dashed line = 95% Confidence interval, plain line = Standard Deviation, square = SMA 2, circle = SMA 3, diamond = ambulant SMA 3, triangle = non ambulant SMA 2, square + circle + triangle = mix phenotypes. Bold font = Median value, Italic = Mean value. Color coding: light blue =  ~10 months from infusion, Red =  ~12 months from infusion, Green =  ~24 months from infusion. Grey shade = SMA 2, White shade = SMA 3, Striped shade = mixed phenotypes. *Mean/median values of the baseline population non excluding drop-outs at T10, 14 or 24 months of follow-up, **mean/median values of the baseline population of both SMA II and III combined

A total of 13 papers reporting nusinersen treated patients and 5 papers reporting studies from untreated cohorts were identified. Two of the 13 studies reporting nusinersen treated patients included both pediatric and adult patients, 9 only adults and 2 only pediatric patients (Fig. 3).

Individual data on RULM were available in one paper (Jochmann 2020), therefore, in order to avoid missing data, we have re-calculated mean change overtime.

With one exception [27], all the manuscripts describing nusinersen treated patients reported an increase on the RULM score and overall the benefit of the treatment resulted to be statistically significant [pooled mean change = 1.11 (95% CI 0.53–1.69)]. For the untreated patients the pooled mean change was 0.47 (95% CI − 0.79–1.74) Pooled mean change across treated and untreated patients was not statistically significant (p = 0.370). Since heterogeneity was considerable (I^2^ = 81.00%) and highly significant we performed a multivariate meta-regression analysis. We found that RULM score change in SMA type 3 patients was significantly lower than in SMA type 2 patients (coefficient (SE)): − 1.00 (0.37), p = 0.007) that in adults the change in the RULM score was significantly lower than pediatric patients (coefficient (SE)): − 1.28 (0.40), p = 0.002. When adjusted for SMA type and age the RULM score was also significantly higher in treated patients than in untreated patients (coefficient (SE)): 1.0 (0.45), p = 0.025). Results remained consistent when we analyzed studies with 10, 12, 14 and 24 months of follow-up, separately (data not shown).

##### Subgroup analysis

###### Adult and pediatric population

Both adult and pediatric populations reported a significant increase on the RULM score (pooled mean change = 0.64 (95% CI 0.27–1.01) and 2.31 (95% CI 0.49–4.14), respectively) with no significance in pooled mean change between the two populations (p = 0.08) (Additional file 4: Fig. S2).

###### SMA type

Both SMA type 2 and SMA type 3 populations reported a significant increase on the RULM score [pooled mean change = 2.05 (95% CI 0.88–3.22) and 0.55 (95% CI 0.12–0.98), respectively] with a difference in pooled mean change between the two populations (p = 0.01).

###### Ambulatory status

Both non-ambulant and ambulant populations reported a significant increase on the RULM score (pooled mean change = 1.16 (95% CI 0.32–2.01) and 0.23 (95% CI − 0.68–1.14) respectively), with a trend of difference in pooled mean change between the two populations (p = 0.14).

##### Minutes-Walk Test (6MWT)

**Fig. 4:**
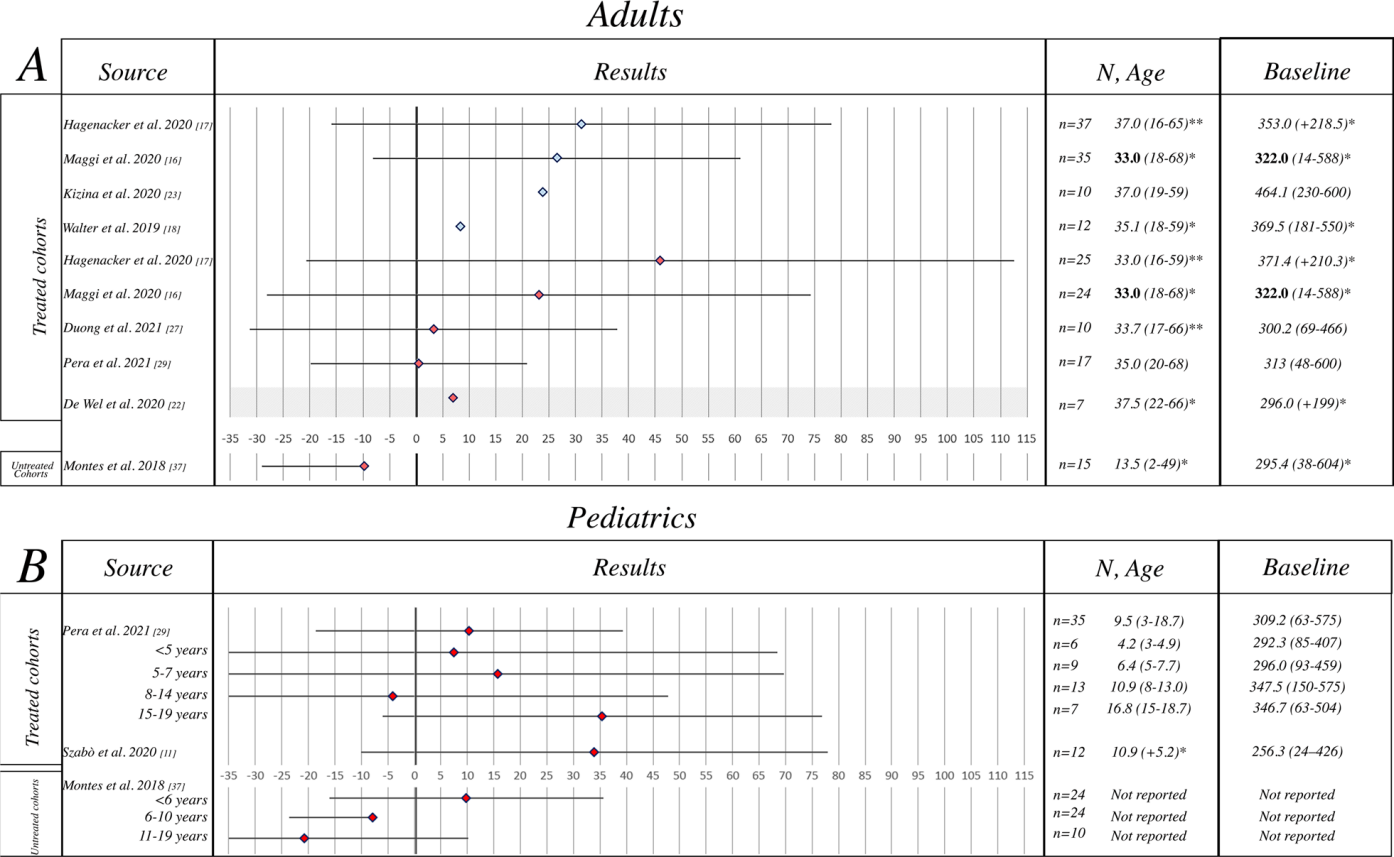
6 min-Walk Test results reporting author, results (mean ± SD), sample size, mean age (years, range/SD), mean baseline 6MWT (range/SD). **a** Adult population, **b** pediatric population. Key to figure: dashed line = 95% Confidence interval, plain line = Standard Deviation. Bold font = Median value, Italic = Mean value. Color coding: light blue =  ~10 months from infusion, Red =  ~12 months from infusion. *Mean/median values of the baseline population non excluding drop-outs at T10, 14 or 24 months of follow-up, **mean/median values of the baseline population of both SMA II and III combined

A total of 8 papers reporting nusinersen and 1 paper reporting studies from untreated cohorts were identified. One of the 8 studies reporting nusinersen treated patients included both pediatric and adults, 6 only adults and 1 only pediatric patient (Fig. 4).

All the manuscripts describing nusinersen treated patients reported an increase on the 6MWT score and overall, the benefit of the treatment resulted to be statistically significant (pooled mean change = 19.80 (95% CI 6.70–32.89). For the untreated patients the pooled mean change was − 8.29 (95% CI − 19.10–2.52). Pooled mean change across treated and untreated patients was statistically significant (p < 0.0001).

Since heterogeneity was high (I^2^ = 88.60%) and highly significant we performed a multivariate meta-regression analysis. When adjusting by age group and treatment, we found that a greater increase in the 6MWT score was associated with the nusinersen treatment (coefficient (standard error (SE)): 27.81 (10.43), p = 0.008), while the 6MWT score change was not significantly associated with age group (pediatric/adult) (p = 0.977).

##### Subgroup analysis

###### Adult and pediatric population

Both adult and pediatric populations reported a significant increase on the 6MWT (20.28 (95% CI 1.17–39.40) and 19.20 (95% CI − 3.20–41.59) respectively, with no statistically significant difference in pooled mean change between the two populations (p = 0.09) (Additional file 4: Fig. S2).

##### Medical Research Council (MRC) Scale for Muscle Strength

###### Adult cohorts

A total of 3 papers reporting nusinersen and 6 paper reporting studies from untreated cohorts were identified. All papers were reporting data from adult population (Table 1).

**Table 1:** MRC results for nusinersen treated and untreated cohorts

Source	SMA TYPE	MRC baseline (mean + SD or mean (range))	Time from infusion/FU timing	N	MRC change
*Nusinersen-treated cohorts*					
De Wel	3 and 4	36.9 + 10.3	14 months	15	Mean: 2.53, 99% CI 0.18–4.88
Walter	3	113.95 + 22.91	10 months	16	Mean: 4.55
Mosche-Lilie	2 and 3	Not reported	12 months	10	Mean %: + 2.13%
*Untreated cohorts*					
Wjngaarde	2A	Not reported	12 months	68	Annual slope: − 0.73, 95% CI − 1.00; − 0.45
Wjngaarde	2B	Not reported	12 months	50	Annual slope: − 0.65, 95% CI − 0.89; − 0.40
Wjngaarde	3A	Not reported	12 months	63	Annual slope: − 0.84, 95% CI − 1.07; − 0.61
Wjngaarde	3B	Not reported	12 months	40	Annual slope: − 0.83, 95% CI − 1.13; − 0.54
Carter GT	2	Mean 2.3 + 0.6*	10 years	18	Decade slope − 0.24 per muscle
Piepers 2008	3B and 4	Mean 294 + 12	Mean 30 months (19–36 months)	9	Mean change: 0
Wadman	1C, 2, 3, 4	Not reported	12 months	180	Annual slope: − 1
Werlauff	2	29% (9–41)	Median 17 years (12–20 years)	21	Annual slope: − 0.22 (Upper limb), CI − 0.39; − 0.02
Otto 2020	2 and 3	142.7 + 41.6	Mean 13.1 months (368–442 days)	9	Mean change: − 1.1

Key to table: *average grade for all muscles groups combined

##### Children’s hospital of Philadelphia—Adult Test of Neuromuscular Disorders (CHOP ATEND)

###### Adult cohorts

One paper was reporting data from the CHOP ATEND in treated patients as exploratory outcome measure. Annual slope of decline for SMA 2 (n = 14) was 3.75 (95% CI − 0.16–7.66), 3.26 (95% CI − 1.34–7.86) for SMA 3 (n = 10) and 3.59 (95% CI 0.67–6.51) for type 2 and 3 combined (n = 24).

##### Motor Function Measurement (MFM)

###### Pediatric cohorts

A total of 2 papers reporting nusinersen and 1 paper reporting studies from untreated cohorts were identified. All papers were reporting data from pediatric population (Table 2).

**Table 2:** MFM results for nusinersen treated and untreated cohorts

Source	SMA TYPE	MFM baseline (mean + SD or mean (range))	Time from infusion/FU timing	n	Mean change mean (SD)	Age at baseline (range)
*Nusinersen-treated cohorts*						
Audic 2020	sma 1–2	45 (10–87)	T12	33	7 (SD not reported)	2–5
Gomez Garcia 2020	sma 1–2	25 + 12	T14	3	5 (SD not reported)	3.5–4.7
Audic 2020	sma 1–2	40 (4–60)	T12	35	3 (SD not reported)	6–17
Gomez Garcia 2020	sma 1–2	37 + 17	T14	13	9 (SD not reported)	6.8–11.5
Gomez Garcia 2020	sma 1–2	34 + 17	T14	16	9 (SD not reported)	3.5–11.5
Audic 2020	sma 1–2	42 (4–87)	T12	68	5 (SD not reported)	2–17
*Untreated cohorts*						
Annoussamy	SMA 2 NON SITTER	not reported	T12	14	− 1.12 (2.29)	2–30
Annoussamy	SMA 2 NON SITTER	not reported	T24	11	− 3.03 (3.77)	2–30
Annoussamy	SMA 2 SITTER	not reported	T12	11	− 2.39 (5.18)	2–30
Annoussamy	SMA 2 SITTER	not reported	T24	4	− 4.95 (8.69)	2–30
Annoussamy	SMA 3 NON AMBULANT	not reported	T12	7	0.35 (3.27)	2–30
Annoussamy	SMA 3 NON AMBULANT	not reported	T24	5	− 0.83 (2.14)	2–30
Annoussamy	SMA 3 AMBULANT	not reported	T12	11	− 1.67 (3.87)	2–30

##### Children’s Hospital of Philadelphia Infant Test of Neuromuscular Disorders (CHOP INTEND)

###### Pediatric cohorts

A total of 2 papers reporting data from nusinersen treated patients were identified. All papers were reporting data from pediatric population. In Audic et al. [12], annual slope of decline for SMA I and 2 combined (n = 14) was 15.1 at 12 months from treatment while in Mendonça et al. [49] was 2.37 (SD:1.13) for SMA 2 and 3 combined (n = 11) at 12 months and 3.4 (95% CI 0–14) at 24 months (n = 7) after treatment started.

##### Hammersmith Infant Neuromuscular Examination (HINE) Section 2: Motor Milestones

###### Pediatric cohorts

A total of 2 papers reporting data from nusinersen treated patients were identified. All papers were reporting data from pediatric population. In Audic et al. [12], annual slope of decline for SMA I and 2 combined (n = 20) was 7.5 at 12 months while in Gomez-Garcia et al. [13] was 1.0 for SMA I and 2 combined (n = 14) at 14 months after treatment started.

Additional file 1: Table S1 reports number of patients reaching clinically meaningful changes on the HFMSE, RULM and 6MWT. Data on other outcome measures were not available.

### Limitations of this study

In this review we focused on functional motor abilities as these were the measures most commonly used (see [50] and [51] for a comprehensive review of strengths and weaknesses/limitations on each scale). Details on respiratory function or safety concerns were not systematically addressed in all the studies reporting motor function and are illustrated in Additional file 2: Table S2. The studies included in this analysis had small number of participants overall or in the subgroups being analyzed. The confidence intervals were often broad, indicating the high variability in these cohorts, and in many cased crossed the zero meridian, which requires a conservating analysis of the data, as was performed here. Unfortunately, since in many studies details on baseline functional status/scores and other variables were missing, we could not perform a detailed statistical analysis or meta-analysis, which could have helped to better understand the possible effect of a number of variables such as age, SMN2 copies or functional ability at baseline. Current registry studies of treated patients may provide more detailed data in the years to follow to better answer these questions.

## Conclusions

In this paper, we reviewed the results in motor function across the published studies reporting real world data in nusinersen treated patients. We only included studies with patients with later onset, classically labelled as type 2 or 3. Even when excluding patients with early onset, the remaining cohort was still very heterogeneous as it included adults and children, type 2 and 3 SMA and ambulant and non-ambulant patients. In order to better understand the possible patterns of efficacy in the absence of a placebo or control group, we also reviewed natural history studies to establish possible differences with data collected in untreated patients using the same measures.

The analysis of the literature resulted into a selection of 19 studies reporting motor function data in nusinersen-treated cohorts, with more studies performed in adult 69% (13/19) than in pediatric patients 21% (4/19), and 10% (2/19) reporting large cohorts including both. Only 6 were focused on a specific SMA type (1 study on type 2, 5 on type 3) while 13 included mixed cohorts, with 7/13 providing details on specific types. In the majority of the studies HFMSE and RULM were the functional measured used to assess efficacy, followed by MFM and MRC and, less frequently, by the HINE2, CHOP INTEND and the recently developed CHOP ATEND. Studies including or focusing on ambulant patients also often used the 6MWT. Not surprisingly the most commonly used tools were those more often selected as outcome measures in clinical trials and in studies on untreated patients. Most studies had a follow up between 10 and 14 months.

The comparison across results was challenging because of differences in the studied cohorts and in the level of details provided in the individual papers, as many studies only provided general results without details on SMA type or ambulatory or baseline functional status. In order to facilitate the comparison across studies and, when available, with data from untreated patients, we reviewed the results focusing first on individual measures and, when available, also reporting information on subgroups according to age, SMA types, and functional abilities.

It is of note that all but one of the studies reported positive changes, irrespective of the SMA type, the age or the functional measure used. These findings are at variance with all the previous studies on untreated cohorts using the same measures showing a tendency to negative changes in both type 2 and 3 pediatric and adult cohorts. Not surprisingly, the positive changes were more obvious in the younger type 2 and 3 patients.

The review of the HFMSE data allowed a more detailed analysis as the scale was used in most real world studies and there were more available published data in untreated patients. All studies in treated patients reported positive HFMSE changes irrespective of the age, SMA type or functional level of the cohorts studied. The positive values were in a relatively narrow range. The only outsiders showing larger improvements were related to small cohorts of few patients with very large standard deviations. Not all the studies had the same duration of follow up, and although there was no obvious difference between the studies reporting 10 month- and those reporting 14 month-follow up, it is of note that in the two largest studies reporting assessments at both time points there was a further increase between 10 and 14 months [16, 17]. Both type 2 and 3 patients showed overall positive changes with type 3 patients having overall larger improvements than type 2 patients. The lack of baseline data in the published cohorts did not allow to better establish possible correlation with the level of function at baseline but as type 2 are known to have very low HFMSE scores and a more severe phenotype after puberty [52] this may reduce the possibility to improve. The availability of HFMSE data in untreated patients also according to age subgroups allowed to establish that, at variance with the treated patients, untreated patients nearly always showed a decline in HFMSE scores. The only exception was in untreated young type 2 and 3 patients who had positive mean 12-month changes before the age of 5 and 7 years respectively [45, 53, 54]. In the same age range the increase in scores in the treated patients was much higher than in untreated ones.

The RULM also showed positive changes in treated patients even though the magnitude of changes was smaller than when using HFMSE. This may be due to a possible ceiling effect that has been previous reported in natural history studies in walkers who have high scores at baseline. Unfortunately, as baseline results were often not available, we are not able to establish possible differences in changes between walkers and sitters [55]. Similar findings were also observed when analyzing the results of the MFM, MRC and 6MWT; each of these also showed positive changes in treated patients. Although for these measures the available data in untreated patients [37–44] did not provide details to allow a direct comparison with the treated patients, all studies in untreated patients also always showed negative changes. Unfortunately, most studies do not provide details on ambulatory status. When available, there was no obvious difference between ambulant and non-ambulant patients.

In conclusion our review highlights that improved motor function can be observed in all the type 2 and 3 cohorts of nusinersen treated patients, in contrast to the negative changes found in studies reporting untreated cohorts. This held true, with very few exceptions, both when considering the overall results of the studies in heterogeneous cohorts or smaller groups subdivided according to age, type or functional status. The efficacy was further confirmed by the evidence that positive changes were observed on all the measures used in the different studies. These real-world results obtained in clinical settings in different countries confirm and expand the positive results reported in more selected cohorts in the clinical trials [7, 56–58]. It is of interest that although most studies had less stringent exclusion criteria than the Cherish study, that excluded patients with contractures, scoliosis and an HFMSE score < 10, the magnitude of improvements in a few real world datasets was still dramatic even in older and more severely affected patients [14, 16–18, 27].

Unfortunately, most studies did not systematically report aspects of fatigue or other aspects of activities of daily living that may have provided a more qualitative assessment to further characterize the possible changes after treatment.

Patients, parents/caregivers, physicians and payers for this expensive drug can benefit from the results of this review that, while confirming the importance of early treatment, suggests a positive response to nusinersen treatment across a broad spectrum of the SMA population.

## Supplementary Information

**Additional file 1. Table S1**: Clinically meaningful change. Key to table: N/A  =  not applicable (e.g. motor scale not used); NR  =  not reported (e.g. study on clinically meaningful change was not performed).

**Additional file 2. Tabls S2**: Safety and respiratory reports. Key to table: * = % calculated on number of infusions, ** = % calculated on number of patients, +  = calculated on number of infusion reported (95 Adverse Events in 25 patients).

**Additional file 3. Fig. S1**: Risk of bias assessment (RoBANS tool).

**Additional file 4. Fig. S2**: Meta-regression analysis results.

## Data Availability

All data generated or analysed during this study are included in this published article.

## Abbreviations

SMA: Spinal muscular atrophy
SMN1: Survival motor neuron 1 gene
FDA: Food and Drug Administration
EMA: European Medicines Agency
SD: Standard deviation
HFMSE: Hammersmith Functional Motor Scale Expanded
RULM: Revised Upper Limb Module
6MWT: 6Minutes-Walk Test
MRC: Medical Research Council Scale for Muscle Strength
CHOP ATEND: Children’s hospital of Philadelphia—Adult Test of Neuromuscular Disorders
MFM: Motor Function Measurement
CHOP INTEND: Children’s Hospital of Philadelphia Infant Test of Neuromuscular Disorders
HINE-2: Hammersmith Infant Neuromuscular Examination Section 2
