# Supplementary material for: Motor function in type 2 and 3 SMA patients treated with Nusinersen: a critical review and meta-analysis
Source: Orphanet J Rare Dis. 2021 Oct 13;16:430. doi: 10.1186/s13023-021-02065-z (PMC8515709; doi:10.1186/s13023-021-02065-z)
Supplement: Supplementary file 1 — Additional file 1. Table S1: Clinically meaningful change. Key to table: N/A = not applicable (e.g. motor scale not used); NR = not reported (e.g. study on clinically meaningful change was not performed). [file 13023_2021_2065_MOESM1_ESM.docx]

| **Study** | **HFMSE** | **RULM** | **6MWT** | **Responders definition:** |
| --- | --- | --- | --- | --- |
| Audic 2020 | N/A | N/A | N/A | N/A |
| Maggi 2020 | *SMA II:*  11% (T10);  20% (T 14) *SMA III sitters:* 40% (T10);  58% (T14) *SMA III walkers:* 43% (T10);  48% (T14) *SMA III tot:*  41% (T10);  52% (T14) | *SMA II:*  56% (T10);  60% (T14) *SMA III sitters:* 39% (T10);  53% (T14) *SMA III walkers:* 11% (T10);  16% (T14) *SMA III tot:*  24% (T10);  32% (T14) | *SMA III:*  46% (T10);  42% (T14) | At least 3 points HFMSE, at least 2 points RULM, at least 30 m 6MWT |
| Szabò 2020 | NR | NR | NR | N/A |
| Konersman 2021 | NR | NR | N/A | N/A |
| Duong 2021 | NR | NR | NR | N/A |
| Kizina 2020 | N/A | N/A | NR | N/A |
| Kessler 2019 | SMA III: 44% (T10) | N/A | N/A | At least 3 points on HFMSE |
| Jockman 2020 | SMA III: 67% (T10) SMA II: 67% (T10) | SMA II: 67%  SMA III: 33% | N/A | At least 3 points on HFMSE or RULM |
| De Wel 2020 | NR | NR | NR | N/A |
| Hagenacker 2020 | SMA II: 30% (T10); 5% (T14) SMA III: 32% (T10); 41% (T14) | NR | NR | At least 3 points on HFMSE |
| Yeo 2020 | SMA III: 50% (T15-21) | SMA III: 33% (T15-21) | N/A | At least 3 points on HFMSE or RULM |
| Osmanovic 2019 | NR | NR | N/A | N/A |
| Walter 2019 | NR | NR | NR | N/A |
| Coratti 2021 | SMA II: 34% (T12) | SMA II: 37% (T12) | N/A | At least 3 points on HFMSE or RULM |
| Mosche-Lilie 2020 | N/A | N/A | N/A | N/A |
| Pera 2021 | NR | NR | NR | N/A |
| Gomez-Garcia 2020 | N/A | N/A | N/A | N/A |
| Mendonca 2020 | NR | N/A | N/A | N/A |
| Veerapandiyan 2020 | N/A | SMA II: 67% (T10) SMA III: 0% | N/A | At least 3 point on RULM |

**Supplementary table 1: Clinically meaningful change**. Key to table: N/A= not applicable (e.g. motor scale not used); NR= not reported (e.g. study on clinically meaningful change was not performed)
