# Supplementary material for: Motor function in type 2 and 3 SMA patients treated with Nusinersen: a critical review and meta-analysis
Source: Orphanet J Rare Dis. 2021 Oct 13;16:430. doi: 10.1186/s13023-021-02065-z (PMC8515709; doi:10.1186/s13023-021-02065-z)
Supplement: Supplementary file 2 — Additional file 2. Tabls S2: Safety and respiratory reports. Key to table: * = % calculated on number of infusions, ** = % calculated on number of patients, + = calculated on number of infusion reported (95 Adverse Events in 25 patients). [file 13023_2021_2065_MOESM2_ESM.docx]

| **Study** | **Respiratory assessments** | **Safety** |
| --- | --- | --- |
| *Szabò 2020* | *Not reported* | Headache 8%* Backache 6%* Vomiting 6%*  *Occasional (% Non-Reported)*  Pneumonia,  Pneumotorax,  Bone Fractures,  Legpain,  Transient Mild Trombocitopenia,  Urinary Tract Infection |
| *Konersman 2020* | *Not reported* | Procedure-related headaches 71%** Procedure-related nausea/vomiting 27%** Pyrexia (all causes) 32%** Procedure-related back pain 44%** Procedure-related meningitis 6%** Elevated PT † 12 88%** Elevated aPTT ‡ 5 69%** Elevated cystatin C-1 5%**  *Hospitalizations (all non-surgical causes):*  69% due to pneumonia 16%**,  Acute Respiratory failure 16%**,  Hypoxia 10%**,  Influenza 10%**,  Otitis media 5%**,  Enterovirus 5%**,  Hematemesis 5%**,  Anxiety/tachycardia 5%**,  Dysphagia 5%**,  Meningitis 6%**, G-tube and Tracheostomy 5%**, Fracture 6%** |
| *Duong 2021* | FVC 0.75%/year MEP 6.38 cmH2O/year MIP -5.50 cmH2O/year | Thrombocytopenia 4.76%** Transient Limb Pain 7.14%**  *Occasional (% Not Reported)*  Injection Site Pain,  Nausea/Vomiting, Lightheadedness,  Anxiety. |
| *Kizina 2020* | *Not reported* | *Not reported* |
| *Kessler 2019* | *Not reported* | *Not reported* |
| *De Wel 2020* | FVC 0.9 L (T14) PEF 0.15 L (T14) | Back Pain 64.3%* Headache 25%* Post Puncture Headache 9.8%* Blood Patch 2.7%* Fatigue 40.2%* Increased Appetite 22.3%* Myalgia 18.8%* Agitation 19.6%* Nausea 12%* Dizziness 8.9%* Proteinuria 5.4%* |
| *Walter 2019* | FVC 5% (T10) | Post puncture headache 10%* Fatigue 0.9%* Back pain 6.5%* |
| *Yeo 2020* | *Not reported* | Post Puncture Headache 67%**  Vertigo 33%** Musculoskeletal And Connective Tissue Problems 67%** Integumentary Problems 33%** |
| *Osmanovic 2019* | *Not reported* | Back Pain 63%** Headache 58%  Nausea 17%** Vertigo 13%** Constipation 8%** Upper Airway Infections 8%** Tachycardia 4%** |
| *Mosche-Lilie 2020* | *Not reported* | Headache 23%** Bacterial Meningitidis 5%**  Pneumonia (Death) 5%** |
| *Maggi, 2020* | FVC 6.47% (T14) | Post puncture headache 37.1%** Lumbar pain 8.6%** Transient worsening of existing hand tremor 2%** Renal colic 8%** |
| *jochmann 2020* | *Not reported* | Headache 29%** Proteinuria 14%** |
| *Hagenacker 2020* | *Not reported* | Headache 11%* Backpain 6%* Nausea 3%* vertigo 1%* Upper airway infections 1%* Constipation 1%* Diffuse pain 0.1%* Bladder disorder not otherwise specified 0.1%* Tinnitus aggravated 0.1%* infections 0.1%* Meningitis aseptic 0.1%* |
| *Mendonca 2020* | *Not reported* | Complication rate 4.2%*  (mainly headache and lower back pain) |
| *Audic 2020* | *Not reported* | 55 technical difficulties^+^ 23 headache^+^ 4 nausea and vomiting^+^ 4 astenia^+^ 2 backpain^+^ 1 fever^+^ |
| *Pera 2021* | *Not reported* | *Not reported* |
| *Coratti 2021* | *Not reported* | *Not reported* |
| *Gomez-Garcia 2020* | *Not reported* | No major event advers side effect |
| *Veerapandiyan 2020* | *Not reported* | Post lumbar puncture headache 9%* Site pain 5.7%* |

**Supplementary table 2: Safety and respiratory reports**. Key to table: *= % calculated on number of infusions, **= % calculated on number of patients, += calculated on number of infusion reported (95 Adverse Events in 25 patients)
