# Supplementary figures and images for: Motor function in type 2 and 3 SMA patients treated with Nusinersen: a critical review and meta-analysis
Source: Orphanet J Rare Dis. 2021 Oct 13;16:430. doi: 10.1186/s13023-021-02065-z (PMC8515709; doi:10.1186/s13023-021-02065-z)

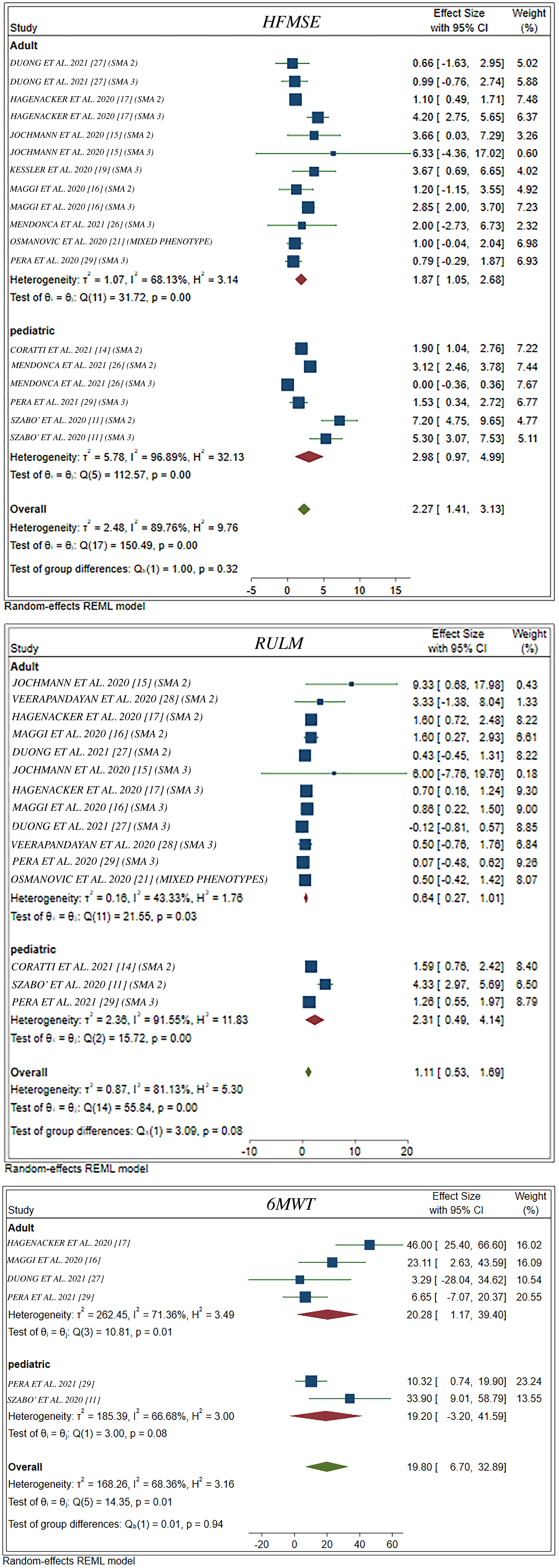

Supplement: Supplementary file 4 — Additional file 4. Fig. S2: Meta-regression analysis results. [file 13023_2021_2065_MOESM4_ESM.tif]
